# Supplementary material for: The infection-tolerant white-footed deermouse tempers interferon responses to endotoxin in comparison to the mouse and rat
Source: eLife. 2024 Jan 9;12:RP90135. doi: 10.7554/eLife.90135 (PMC10945503; doi:10.7554/eLife.90135)
Supplement: Supplementary file 1. — The list includes the 113 coding sequences listed in Methods, as well as endogenous retrovirus (ERV) env transcripts and ERV gag-pol transcripts. The values are mean unique reads with asymmetric 95% confidence intervals for a given gene transcript normalized for reads for Ptprc transcripts for a sample. Each species is analyzed separately with respect to LPS-treated and control animals. For cross-species comparisons the fold-change (FC) differences between control and LPS-treated animals were individually determined. For a summary representation of the differences between the two species in responses to LPS, the ratios of P. leucopus to M. musculus FC values were calculated. These are schematically displayed as a color-coded heat-map as well. [file elife-90135-supp1.docx]

| Supplementary File 1. Targeted RNA-seq of blood for 115 selected genes of *Peromyscus leucopus* and *Mus musculus* 4 hours after intraperitoneal injection of lipopolysaccharide (LPS) or saline control | | | | | | | | | | | | |
| --- | --- | --- | --- | --- | --- | --- | --- | --- | --- | --- | --- | --- |
| Gene * | Peromyscus/ Mus LPS/control Heatmap† | Mus control mean (95% CI)‡ | Mus LPS mean (95% CI) | Mus FC LPS vs control** | Mus FDR p value** | Peromyscus control mean (95% CI) | Peromyscus LPS mean (95% CI) | Peromyscus FC LPS v control | Peromyscus FDR p value | Peromyscus v Mus control FC | Peromyscus v Mus LPS FC | Peromyscus/ Mus LPS/control† |
| *Ibsp* | >100 | 0.51 (0.28-0.93) | 5.99 (2.04-17.6) | 11.7 | 2E-03 | 0.17 (0.06-0.47) | 471 (305-728) | 2720 | 3E-10 | 0.34 | 78.6 | 233 |
| *Slpi* |  | 42.0 (32.6-54.0) | 93.2 (44.5-195) | 2.22 | 7E-02 | 1.94 (0.96-3.94) | 921 (636-1333) | 474 | 1E-10 | 0.05 | 9.88 | 213 |
| *Steap1* | 10-100 | 0.13 (0.10-0.16) | 0.58 (0.21-1.60) | 4.52 | 1E-02 | 0.09 (0.05-0.17) | 31.7 (16.3-61.4) | 335 | 1E-09 | 0.73 | 54.3 | 74.2 |
| *Saa3* |  | 2.82 (0.89-8.95) | 458 (165-1267) | 162 | 7E-06 | 0.21 (0.12-0.37) | 2052 (1273-3306) | 9934 | 2E-13 | 0.07 | 4.48 | 61.2 |
| *Lpo* |  | 0.13 (0.09-0.20) | 0.37 (0.19-0.72) | 2.78 | 2E-02 | 0.03 (0.03-0.04) | 4.10 (1.44-11.7) | 118 | 1E-07 | 0.26 | 11.2 | 42.4 |
| *Mmp8* |  | 67.4 (34.3-133) | 298 (187-477) | 4.43 | 3E-03 | 10.9 (4.26-27.8) | 1823 (1311-2534) | 168 | 3E-08 | 0.16 | 6.11 | 37.9 |
| *Lrg* |  | 40.8 (22.1-75.3) | 107 (52.2-219) | 2.62 | 7E-02 | 8.01 (2.98-21.5) | 353 (268-464) | 44.1 | 2E-06 | 0.20 | 3.30 | 16.8 |
| *Mmp9* |  | 222 (147-335) | 153 (90.1-258) | 0.69 | 3E-01 | 154 (96.8-245) | 1258 (903-1753) | 8.17 | 2E-06 | 0.69 | 8.25 | 11.9 |
| *Csf3* |  | 0.14 (0.10-0.19) | 7.85 (3.07-20.1) | 57.1 | 6E-07 | 0.04 (0.03-0.05) | 24.5 (8.95-67.1) | 648 | 2E-09 | 0.27 | 3.12 | 11.4 |
| *Serpine1* |  | 0.51 (0.27-0.93) | 11.7 (3.04-45.2) | 23.2 | 9E-04 | 0.14 (0.06-0.32) | 32.8 (4.82-223) | 239 | 9E-05 | 0.27 | 2.80 | 10.3 |
| *Fpr2* | 4.0-9.99 | 36.9 (25.8-52.7) | 246 (168-362) | 6.68 | 2E-06 | 8.74 (6.48-11.8) | 535 (399-717) | 61.2 | 5E-12 | 0.24 | 2.17 | 9.16 |
| *Cxcl3* |  | 0.32 (0.17-0.59) | 19.6 (12.3-31.3) | 60.8 | 2E-08 | 0.09 (0.04-0.21) | 47.2 (15.9-140) | 523 | 2E-07 | 0.28 | 2.41 | 8.59 |
| *Retn* |  | 0.35 (0.18-0.68) | 0.45 (0.26-0.77) | 1.28 | 6E-01 | 3.13 (1.80-5.46) | 31.8 (22.0-45.9) | 10.1 | 4E-06 | 8.96 | 71.1 | 7.93 |
| *Csf3r* |  | 669 (455-982) | 302 (221-411) | 0.45 | 7E-03 | 423 (318-563) | 1445 (1024-2040) | 3.42 | 6E-05 | 0.63 | 4.79 | 7.58 |
| *Fcgr3* |  | 45.7 (31.7-65.8) | 70.1 (35.1-140) | 1.53 | 3E-01 | 44.7 (34.1-58.6) | 409 (338-497) | 9.17 | 1E-09 | 0.98 | 5.84 | 5.97 |
| *Ptx* |  | 0.24 (0.17-0.33) | 4.06 (0.94-17.6) | 17.0 | 2E-03 | 0.05 (0.03-0.09) | 4.88 (1.10-21.6) | 92.2 | 4E-05 | 0.22 | 1.20 | 5.42 |
| *Fcgr2a* |  | 78.8 (59.1-105) | 149 (93.8-237) | 1.89 | 4E-02 | 75.0 (62.0-90.8) | 766 (597-983) | 10.2 | 2E-10 | 0.95 | 5.14 | 5.40 |
| *Cd177* |  | 25.0 (11.1-56.1) | 158 (80.2-310) | 6.31 | 4E-03 | 4.29 (2.12-8.69) | 144 (107-194) | 33.5 | 1E-07 | 0.17 | 0.91 | 5.31 |
| *Ncf4* |  | 134 (112-160) | 204 (172-242) | 1.52 | 5E-03 | 49.1 (38.4-62.9) | 317 (258-389) | 6.46 | 5E-09 | 0.37 | 1.55 | 4.24 |
| *Il1b* |  | 158 (102-244) | 464 (341-631) | 2.94 | 1E-03 | 230 (146-362) | 2845 (1998-4052) | 12.4 | 2E-07 | 1.45 | 6.14 | 4.22 |
| *Fcgr2b* |  | 32.7 (27.4-39.0) | 111 (78.2-156) | 3.38 | 1E-05 | 24.9 (22.4-27.8) | 352 (302-409) | 14.1 | 2E-14 | 0.76 | 3.18 | 4.17 |
| *Hk3* | 2.00-3.99 | 35.8 (25.7-50.0) | 63.6 (46.6-86.8) | 1.78 | 3E-02 | 85.5 (66.8-109) | 602 (468-773) | 7.04 | 1E-08 | 2.39 | 9.46 | 3.96 |
| *Fgr* |  | 138 (110-174) | 161 (125-207) | 1.17 | 4E-01 | 251 (205-308) | 1131 (987-1295) | 4.50 | 3E-09 | 1.82 | 7.02 | 3.85 |
| *Tlr4* |  | 11.9 (8.62-16.4) | 11.9 (6.23-22.8) | 1.00 | 1E+00 | 29.7 (22.9-38.6) | 113 (90.7-141) | 3.81 | 1E-06 | 2.50 | 9.49 | 3.80 |
| *Glrx* |  | 54.8 (43.1-69.7) | 122 (65.4-227) | 2.22 | 4E-02 | 12.4 (11.1-13.9) | 88.4 (71.6-109) | 7.11 | 5E-11 | 0.23 | 0.73 | 3.20 |
| *Slc11a1* |  | 54.9 (41.6-72.5) | 75.3 (60.0-94.6) | 1.37 | 1E-01 | 104 (88.0-123) | 424 (308-582) | 4.07 | 1E-06 | 1.89 | 5.62 | 2.97 |
| *Ccl3* |  | 3.07 (2.20-4.30) | 227 (161-320) | 73.9 | 2E-11 | 0.26 (0.14-0.48) | 54.6 (33.2-89.8) | 213 | 1E-09 | 0.08 | 0.24 | 2.88 |
| *Tlr1* |  | 71.8 (63.3-81.5) | 52.2 (37.4-72.8) | 0.73 | 1E-01 | 36.0 (29.8-43.4) | 73.3 (61.6-87.1) | 2.04 | 5E-05 | 0.50 | 1.40 | 2.81 |
| *Hmox1* |  | 16.0 (12.5-20.6) | 27.8 (13.7-56.7) | 1.74 | 2E-01 | 17.4 (14.9-20.4) | 78.2 (51.5-119) | 4.49 | 6E-06 | 1.09 | 2.81 | 2.58 |
| *Tnfrsf1a* |  | 70.8 (57.2-87.7) | 69.3 (45.5-106) | 0.98 | 9E-01 | 44.6 (34.1-58.4) | 105 (89.0-123) | 2.34 | 6E-05 | 0.63 | 1.51 | 2.40 |
| *Olfm4* |  | 7.41 (3.43-16.0) | 162 (98.8-266) | 21.9 | 6E-06 | 3.56 (1.96-6.44) | 181 (101-326) | 51.0 | 1E-07 | 0.48 | 1.12 | 2.33 |
| *Sod2* |  | 58.1 (48.3-69.9) | 412 (262-650) | 7.10 | 8E-07 | 124 (80.5-192) | 2030 (1666-2475) | 16.3 | 5E-09 | 2.14 | 4.92 | 2.30 |
| *Il2ra* | 1.51-1.99 | 12.3 (3.95-38.3) | 8.23 (3.06-22.2) | 0.67 | 6E-01 | 4.33 (3.80-4.94) | 5.77 (4.38-7.61) | 1.33 | 9E-02 | 0.35 | 0.70 | 1.99 |
| *Lcn2* |  | 29.6 (14.9-58.9) | 2797 (1832-4271) | 94.5 | 1E-08 | 25.2 (11.4-55.9) | 4571 (3268-6393) | 181 | 3E-09 | 0.85 | 1.63 | 1.92 |
| *Sting* |  | 51.2 (42.7-61.3) | 47.8 (38.5-59.3) | 0.93 | 7E-01 | 181 (160-204) | 317 (277-363) | 1.76 | 2E-05 | 3.53 | 6.64 | 1.88 |
| *Jak2* |  | 166 (150-185) | 355 (267-472) | 2.13 | 2E-04 | 6.02 (5.43-6.66) | 23.4 (16.5-33.2) | 3.89 | 2E-06 | 0.04 | 0.07 | 1.82 |
| *Lrrk2* |  | 93.9 (82.2-107) | 83.5 (66.1-105) | 0.89 | 4E-01 | 153 (125-188) | 236 (163-340) | 1.54 | 6E-02 | 1.63 | 2.82 | 1.73 |
| *Il1rn* |  | 18.6 (12.1-28.6) | 325 (185-572) | 17.5 | 7E-07 | 17.4 (13.0-23.3) | 523 (408-670) | 30.0 | 2E-11 | 0.94 | 1.61 | 1.72 |
| *Csf1* |  | 8.18 (5.53-12.1) | 17.5 (12.0-25.5) | 2.14 | 2E-02 | 3.32 (2.43-4.55) | 12.1 (8.04-18.1) | 3.63 | 1E-04 | 0.41 | 0.69 | 1.70 |
| *Cfb* |  | 6.28 (4.26-9.25) | 111 (46.0-269) | 17.7 | 2E-05 | 7.93 (3.52-17.9) | 225 (140-362) | 28.4 | 3E-06 | 1.26 | 2.03 | 1.60 |
| *Itgam* |  | 265 (207-339) | 361 (283-461) | 1.37 | 1E-01 | 183 (135-250) | 396 (278-564) | 2.16 | 5E-03 | 0.69 | 1.10 | 1.58 |
| *Timp1* |  | 108 (94.0-124) | 192 (117-316) | 1.78 | 5E-02 | 102 (91.0-114) | 272 (226-326) | 2.67 | 1E-07 | 0.94 | 1.41 | 1.50 |
| *Socs3* | 0.67-1.50 | 19.9 (12.8-30.8) | 254 (213-304) | 12.8 | 2E-08 | 16.8 (12.1-23.4) | 304 (224-413) | 18.1 | 1E-09 | 0.84 | 1.20 | 1.42 |
| *Il6* |  | 0.27 (0.17-0.42) | 9.27 (5.27-16.3) | 34.5 | 5E-08 | 0.17 (0.09-0.33) | 7.77 (1.74-34.6) | 45.5 | 3E-04 | 0.64 | 0.84 | 1.32 |
| *Ltf* |  | 10.5 (5.82-18.8) | 824 (445-1526) | 78.8 | 3E-08 | 28.5 (12.0-68.0) | 2930 (2069-4151) | 103 | 5E-08 | 2.73 | 3.56 | 1.30 |
| *S100a9* |  | 912 (539-1543) | 4540 (2426-8497) | 4.98 | 2E-03 | 2036 (1240-3344) | 12972 (10079-16695) | 6.37 | 6E-06 | 2.23 | 2.86 | 1.28 |
| *Cxcl1* |  | 0.13 (0.09-0.19) | 20.3 (9.03-45.7) | 158 | 9E-09 | 0.06 (0.03-0.13) | 12.3 (3.08-49.0) | 198 | 6E-06 | 0.48 | 0.60 | 1.25 |
| *Il10* |  | 0.11 (0.10-0.13) | 3.57 (1.86-6.87) | 31.7 | 3E-08 | 0.09 (0.05-0.15) | 3.53 (1.13-11.0) | 39.6 | 3E-05 | 0.79 | 0.99 | 1.25 |
| *Gapdh* |  | 646 (591-707) | 1014 (558-1845) | 1.57 | 2E-01 | 667 (590-754) | 1295 (923-1818) | 1.94 | 2E-03 | 1.03 | 1.28 | 1.24 |
| *Csf1r* |  | 233 (201-270) | 47.3 (31.7-70.7) | 0.20 | 2E-06 | 320 (262-390) | 78.3 (44.7-137) | 0.24 | 2E-04 | 1.37 | 1.66 | 1.21 |
| *Nfkb1* |  | 149 (133-168) | 336 (283-399) | 2.25 | 1E-06 | 118 (114-122) | 300 (209-431) | 2.54 | 1E-04 | 0.79 | 0.89 | 1.13 |
| *Nr3c1* |  | 124 (105-148) | 119 (101-139) | 0.95 | 7E-01 | 47.0 (41.5-53.1) | 49.9 (38.3-65.2) | 1.06 | 7E-01 | 0.38 | 0.42 | 1.11 |
| *Akt1* |  | 98.1 (82.8-116) | 68.1 (54.1-85.7) | 0.69 | 3E-02 | 148 (133-164) | 113 (88.8-144) | 0.76 | 6E-02 | 1.51 | 1.66 | 1.10 |
| *Pkm* |  | 538 (469-618) | 884 (506-1544) | 1.64 | 1E-01 | 417 (364-479) | 741 (558-983) | 1.77 | 2E-03 | 0.78 | 0.84 | 1.08 |
| *Il4ra* |  | 91.2 (80.2-104) | 247 (198-309) | 2.71 | 1E-06 | 51.7 (47.2-56.7) | 150 (122-184) | 2.90 | 8E-08 | 0.57 | 0.61 | 1.07 |
| *Fcer2* |  | 104 (70.7-153) | 15.7 (10.0-24.5) | 0.15 | 1E-05 | 55.7 (47.6-65.1) | 8.93 (4.96-16.1) | 0.16 | 2E-05 | 0.54 | 0.57 | 1.06 |
| *Camp* |  | 3.12 (1.76-5.54) | 285 (145-558) | 91.2 | 3E-08 | 5.42 (2.26-13.0) | 524 (354-776) | 96.7 | 8E-08 | 1.74 | 1.84 | 1.06 |
| *Hif1a* |  | 53.4 (46.5-61.5) | 191 (144-254) | 3.58 | 8E-07 | 83.6 (77.0-90.8) | 299 (203-440) | 3.58 | 1E-05 | 1.56 | 1.57 | 1.00 |
| *Padi4* |  | 15.9 (8.70-29.0) | 41.2 (22.3-76.0) | 2.59 | 5E-02 | 44.6 (34.1-58.4) | 105 (89.0-123) | 2.34 | 6E-05 | 2.81 | 2.54 | 0.90 |
| *Bcl3* |  | 21.1 (14.8-30.0) | 209 (168-260) | 9.92 | 1E-08 | 18.9 (15.1-23.8) | 167 (127-219) | 8.80 | 3E-09 | 0.90 | 0.80 | 0.89 |
| *Mt2* |  | 0.38 (0.19-0.77) | 89.1 (33.8-235) | 235 | 1E-07 | 0.31 (0.13-0.75) | 61.2 (20.5-182) | 199 | 2E-06 | 0.81 | 0.69 | 0.84 |
| *Myd88* |  | 50.8 (39.5-65.4) | 127 (96.0-168) | 2.50 | 2E-04 | 51.5 (46.6-56.8) | 108 (99.7-116) | 2.09 | 5E-09 | 1.01 | 0.85 | 0.84 |
| *Thy1* |  | 35.0 (22.6-54.2) | 25.2 (13.3-48.0) | 0.72 | 5E-01 | 503 (457-553) | 297 (240-368) | 0.59 | 4E-04 | 14.4 | 11.8 | 0.82 |
| *Jun* |  | 31.4 (23.9-41.2) | 157 (105-234) | 5.01 | 6E-06 | 6.02 (5.43-6.66) | 23.4 (16.5-33.2) | 3.89 | 2E-06 | 0.19 | 0.15 | 0.78 |
| *Icam* |  | 24.1 (20.3-28.6) | 301 (257-352) | 12.5 | 3E-12 | 12.6 (10.5-15.0) | 121 (67.3-217) | 9.61 | 2E-06 | 0.52 | 0.40 | 0.77 |
| *Cx3cr1* |  | 27.9 (21.8-35.7) | 3.46 (1.99-6.00) | 0.12 | 4E-06 | 26.1 (19.0-35.9) | 2.45 (1.44-4.15) | 0.09 | 1E-06 | 0.94 | 0.71 | 0.76 |
| *Cxcl2* |  | 0.16 (0.09-0.26) | 119 (76.0-186) | 762 | 6E-12 | 0.10 (0.04-0.26) | 60.2 (19.6-185) | 574 | 2E-07 | 0.67 | 0.51 | 0.75 |
| *Akt2* |  | 138 (117-163) | 275 (182-415) | 1.99 | 9E-03 | 59.4 (57.1-61.7) | 87.6 (71.5-107) | 1.47 | 2E-03 | 0.43 | 0.32 | 0.74 |
| *Jak1* |  | 415 (386-447) | 623 (467-830) | 1.50 | 2E-02 | 256 (232-281) | 277 (214-359) | 1.09 | 6E-01 | 0.61 | 0.45 | 0.72 |
| ERV *gag-pol* |  | 3210 (2406-4283) | 13633 (10083-18433) | 4.3 | 4E-06 | 233 (195-278) | 692 (548-873) | 2.97 | 2E-06 | 0.07 | 0.05 | 0.70 |
| *Pbib* |  | 93.7 (82.3-107) | 97.2 (58.5-161) | 1.04 | 9E-01 | 143 (134-153) | 102 (71.2-147) | 0.71 | 9E-02 | 1.53 | 1.05 | 0.69 |
| *Nox1* | 0.50-0.66 | 0.14 (0.11-0.18) | 0.98 (0.65-1.48) | 7.01 | 8E-07 | 10.1 (8.12-12.5) | 45.6 (34.4-60.5) | 4.53 | 3E-07 | 71.8 | 46.4 | 0.65 |
| *Tnf* |  | 2.99 (2.26-3.96) | 19.5 (14.0-27.1) | 6.52 | 3E-07 | 0.76 (0.42-1.40) | 3.10 (1.62-5.95) | 4.06 | 7E-03 | 0.26 | 0.16 | 0.62 |
| *Tlr2* |  | 26.1 (18.1-37.5) | 165 (126-215) | 6.31 | 6E-07 | 50.8 (37.2-69.5) | 194 (128-294) | 3.83 | 1E-04 | 1.95 | 1.18 | 0.61 |
| *Cd8* |  | 23.8 (19.6-29.0) | 17.3 (13.6-22.1) | 0.73 | 7E-02 | 52.6 (41.6-66.5) | 21.3 (10.2-44.7) | 0.41 | 4E-02 | 2.21 | 1.23 | 0.56 |
| *Stat1* |  | 160 (132-193) | 449 (247-814) | 2.81 | 6E-03 | 256 (221-297) | 387 (323-465) | 1.51 | 3E-03 | 1.60 | 0.86 | 0.54 |
| *Mapk1* |  | 71.1 (65.5-77.0) | 92.6 (65.0-132) | 1.30 | 2E-01 | 67.5 (63.9-71.3) | 44.2 (37.4-52.3) | 0.65 | 2E-04 | 0.95 | 0.48 | 0.50 |
| *Arg1* |  | 1.12 (0.36-3.42) | 18.4 (5.99-56.7) | 16.5 | 4E-03 | 261 (154-441) | 2161 (1709-2732) | 8.28 | 2E-06 | 234 | 117 | 0.50 |
| *Ccl2* |  | 0.16 (0.10-0.27) | 12.7 (4.87-33.0) | 77.7 | 8E-07 | 0.19 (0.07-0.50) | 7.42 (3.23-17.0) | 38.5 | 3E-05 | 1.18 | 0.58 | 0.50 |
| *Rigi* (*Ddx58*) | 0.11-0.49 | 47.3 (41.9-53.3) | 293 (175-491) | 6.20 | 0.00 | 26.6 (20.5-34.4) | 98.9 (76.9-127) | 3.72 | 0.00 | 0.60 | 0.29 | 0.49 |
| *MT-Co1* |  | 10015 (7976-12576) | 13316 (6322-28047) | 1.33 | 5E-01 | 6608 (4976-8775) | 4164 (2563-6765) | 0.63 | 1E-01 | 0.66 | 0.31 | 0.47 |
| *Cd4* |  | 55.6 (45.7-67.6) | 27.5 (19.6-38.8) | 0.50 | 4E-03 | 88.5 (75.5-104) | 19.9 (10.4-38.3) | 0.23 | 5E-04 | 1.59 | 0.72 | 0.45 |
| *Socs1* |  | 0.82 (0.59-1.13) | 45.4 (25.6-80.4) | 55.6 | 3E-09 | 0.74 (0.53-1.04) | 18.4 (9.19-36.9) | 24.8 | 5E-07 | 0.91 | 0.41 | 0.45 |
| *Cd3d* |  | 81.9 (71.8-93.4) | 46.7 (32.8-66.5) | 0.57 | 1E-02 | 77.1 (64.1-92.7) | 18.3 (8.91-37.6) | 0.24 | 2E-03 | 0.94 | 0.39 | 0.42 |
| *Mtor* |  | 27.9 (24.3-31.9) | 40.2 (34.9-46.3) | 1.44 | 2E-03 | 62.1 (55.6-69.4) | 36.3 (27.4-48.1) | 0.58 | 3E-03 | 2.23 | 0.90 | 0.41 |
| *Il18* |  | 1.78 (1.18-2.70) | 4.24 (2.84-6.32) | 2.38 | 1E-02 | 4.89 (4.22-5.67) | 4.15 (2.40-7.18) | 0.85 | 6E-01 | 2.74 | 0.98 | 0.36 |
| *Stat2* |  | 41.0 (36.6-45.9) | 229 (144-364) | 5.59 | 2E-06 | 89.1 (75.3-105) | 177 (153-206) | 1.99 | 2E-05 | 2.17 | 0.77 | 0.36 |
| *Il7r* |  | 36.8 (29.2-46.3) | 39.6 (28.4-55.1) | 1.08 | 7E-01 | 59.2 (50.5-69.3) | 22.3 (15.5-32.0) | 0.38 | 2E-04 | 1.61 | 0.56 | 0.35 |
| *Tgfb* |  | 0.13 (0.09-0.19) | 8.02 (3.82-16.8) | 60.6 | 4E-08 | 1.08 (0.78-1.50) | 22.1 (11.5-42.5) | 20.4 | 5E-07 | 8.18 | 2.76 | 0.34 |
| *Acod1* |  | 1.20 (0.60-2.37) | 388 (289-521) | 324 | 2E-10 | 2.64 (1.72-4.05) | 276 (202-378) | 105 | 2E-11 | 2.21 | 0.71 | 0.32 |
| *Csf2* |  | 0.16 (0.11-0.23) | 3.94 (2.09-7.43) | 24.9 | 3E-07 | 0.05 (0.03-0.07) | 0.33 (0.11-0.98) | 7.10 | 4E-03 | 0.29 | 0.08 | 0.28 |
| *Stat4* |  | 55.4 (50.4-60.8) | 110 (95.3-128) | 1.99 | 8E-07 | 26.4 (21.8-32.0) | 14.4 (10.4-19.9) | 0.55 | 6E-03 | 0.48 | 0.13 | 0.27 |
| *Dhx58* |  | 11.3 (9.76-13.2) | 156 (77.1-317) | 13.8 | 2E-06 | 20.2 (14.3-28.4) | 73.1 (55.9-95.5) | 3.63 | 3E-05 | 1.78 | 0.47 | 0.26 |
| *Isg15* |  | 81.5 (65.6-101) | 600 (219-1646) | 7.37 | 2E-03 | 183 (104-322) | 342 (175-668) | 1.87 | 2E-01 | 2.24 | 0.57 | 0.25 |
| *Oas1* |  | 10.6 (8.90-12.6) | 106 (49.4-225) | 9.98 | 3E-05 | 81.1 (51.9-127) | 191 (127-285) | 2.35 | 1E-02 | 7.67 | 1.81 | 0.24 |
| *Cgas* |  | 1.55 (1.19-2.02) | 17.3 (14.3-21.0) | 11.2 | 4E-10 | 5.53 (4.52-6.77) | 13.7 (10.8-17.4) | 2.47 | 3E-05 | 3.57 | 0.79 | 0.22 |
| *Gbp4* |  | 86.1 (73.9-100) | 795 (523-1208) | 9.23 | 4E-08 | 81.5 (70.5-94.1) | 153 (112-209) | 1.88 | 2E-03 | 0.95 | 0.19 | 0.20 |
| *Il12* |  | 2.85 (2.19-3.71) | 11.7 (7.75-17.8) | 4.12 | 4E-05 | 0.30 (0.17-0.53) | 0.25 (0.14-0.43) | 0.82 | 6E-01 | 0.11 | 0.02 | 0.20 |
| ERV *env* |  | 913 (661-1261) | 3398 (2240-5155) | 3.7 | 2E-04 | 48.1 (42.9-53.9) | 33.1 (25.7-47.5) | 0.69 | 2E-02 | 0.05 | 0.01 | 0.18 |
| *Irf7* |  | 43.5 (34.1-55.5) | 528 (276-1010) | 12.1 | 2E-06 | 145 (83.0-253) | 296 (186-469) | 2.04 | 7E-02 | 3.33 | 0.56 | 0.17 |
| *Fos* |  | 16.6 (11.9-23.2) | 17.3 (9.38-31.8) | 1.04 | 9E-01 | 20.2 (14.5-28.0) | 2.39 (1.61-3.55) | 0.12 | 5E-07 | 1.21 | 0.14 | 0.11 |
| *Ifih1* | 0.01-0.10 | 8.39 (6.56-10.7) | 181 (82.4-395) | 21.5 | 2E-06 | 43.3 (35.5-52.8) | 92.1 (75.8-112) | 2.13 | 6E-05 | 5.16 | 0.51 | 0.10 |
| *Cxcl10* |  | 0.35 (0.21-0.59) | 231 (81.4-655) | 659 | 1E-08 | 0.26 (0.12-0.59) | 16.2 (5.83-44.9) | 61.7 | 1E-05 | 0.75 | 0.07 | 0.094 |
| *Mpo* |  | 0.33 (0.19-0.57) | 19.5 (8.56-44.6) | 59.9 | 6E-07 | 1.73 (0.71-4.26) | 7.91 (2.61-24.0) | 4.56 | 5E-02 | 5.32 | 0.41 | 0.076 |
| *Myc* |  | 22.4 (18.0-27.7) | 294 (209-413) | 13.1 | 3E-09 | 41.6 (35.9-48.3) | 41.4 (25.2-68.1) | 1.00 | 1E+00 | 1.86 | 0.14 | 0.076 |
| *Ngp* |  | 7.82 (4.03-15.2) | 1071 (580-1977) | 137 | 1E-08 | 130 (77.8-216) | 1275 (1022-1589) | 9.83 | 5E-07 | 16.6 | 1.19 | 0.072 |
| *Ifit1* |  | 10.7 (8.18-13.9) | 309 (96.6-991) | 29.0 | 5E-05 | 53.1 (32.8-85.8) | 96.7 (59.0-158) | 1.82 | 1E-01 | 4.98 | 0.31 | 0.063 |
| *Mx2* |  | 6.29 (5.53-7.16) | 270 (158-460) | 42.9 | 1E-09 | 198 (121-323) | 476 (300-757) | 2.41 | 2E-02 | 31.5 | 1.76 | 0.056 |
| *Cd69* |  | 14.1 (11.5-17.3) | 203 (155-266) | 14.4 | 1E-10 | 39.3 (34.6-44.6) | 31.1 (17.3-55.9) | 0.79 | 5E-01 | 2.78 | 0.15 | 0.055 |
| *Ccl4* |  | 1.84 (1.10-3.09) | 217 (126-376) | 118 | 3E-09 | 117 (75.4-181) | 740 (487-1126) | 6.33 | 2E-05 | 63.5 | 3.40 | 0.054 |
| *Gzmb* |  | 14.7 (9.75-22.1) | 1063 (603-1873) | 72.4 | 3E-09 | 28.8 (15.8-52.6) | 103 (56.2-188) | 3.57 | 1E-02 | 1.96 | 0.10 | 0.049 |
| *Tnfrsf9* |  | 0.64 (0.46-0.89) | 97.5 (47.3-201) | 152 | 3E-09 | 0.76 (0.42-1.40) | 3.10 (1.62-5.95) | 4.06 | 7E-03 | 1.19 | 0.03 | 0.027 |
| *Cd14* |  | 44.5 (25.5-77.7) | 667 (408-1091) | 15.0 | 2E-06 | 88.5 (75.5-104) | 19.9 (10.4-38.3) | 0.23 | 5E-04 | 1.99 | 0.03 | 0.015 |
| *Nos2* |  | 0.13 (0.10-0.17) | 58.9 (22.1-157) | 459 | 4E-09 | 0.04 (0.03-0.05) | 0.25 (0.09-0.69) | 6.16 | 3E-03 | 0.31 | 0.00 | 0.013 |
| *Ifng* | <0.01 | 0.58 (0.33-1.02) | 438 (178-1080) | 751 | 3E-09 | 0.15 (0.08-0.29) | 0.51 (0.14-1.89) | 3.30 | 1E-01 | 0.26 | 0.00 | 0.004 |
| * Gene: Mus musculus reference genome GenBank name (other name in literature). The list includes the 113 coding sequences listed in Methods and two endogenous retrovius (ERV) proteins, Env and Gag-pol. | | | | | | | | | | | | |
| † Heatmap (brown-gray-blue scale) of "Peromyscus v Mus LPS/control" values (last column). This is ratio of "Peromyscus v Mus LPS FC" (2nd column from right) to "Peromyscus v Mus control FC" (3rd column from right). The range of ratios for each color shading is indicated in the first cell for a given shading. | | | | | | | | | | | | |
| ‡ Mean unique reads for given gene normalized for reads for the transcript of *Ptprc* (Cd45) for a sample. The 95% confidence intervals (CI) are asymmetric. Actual [gene]/Ptprc ratios are x 10^-3^. | | | | | | | | | | | | |
| ** Other abbreviations: Peromyscus, *Peromyscus leucopus*; Mus*, Mus musculus*; ERV, endogenous retrovirus; Env, envelope protein; Gag-pol, *gag-pol* polyprotein, FC, fold-change; FDR, false discovery rate | | | | | | | | | | | | |
